# Development and Validation of an ^18^F-Fluorodeoxyglucose Positron Emission Tomography-Computed Tomography–Based Imaging Score to Predict 12-Week Life Expectancy in Advanced Chemorefractory Colorectal Cancer

**DOI:** 10.1200/CCI-24-00207

**Published:** 2025-04-30

**Authors:** Zelda Paquier, Jennifer Dhont, Thomas Guiot, Hugo Levillain, Gabriela Critchi, Rita Saude Conde, Francesco Sclafani, Patrick Flamen, Nick Reynaert, Erwin Woff, Alain Hendlisz

**Affiliations:** ^1^ Radiophysics and MRI Physics Laboratory, Université Libre De Bruxelles (ULB), Brussels, Belgium; ^2^ Department of Medical Physics, Institut Jules Bordet, Hôpital Universitaire de Bruxelles (HUB), Université libre de Bruxelles (ULB), Brussels, Belgium; ^3^ Department of Gastrointestinal Oncology, Institut Jules Bordet, Hôpital Universitaire de Bruxelles (HUB), Université libre de Bruxelles (ULB), Brussels, Belgium; ^4^ Department of Nuclear Medicine, Institut Jules Bordet, Hôpital Universitaire de Bruxelles (HUB), Université libre de Bruxelles (ULB), Brussels, Belgium

## Abstract

**PURPOSE:**

Managing chemorefractory metastatic colorectal cancer (mCRC) requires a meticulous equilibrium between the efficacy and toxicity of interventions, a task compounded by the constrained life expectancy of the patient. While existing prognostic tools, such as the Colon Life nomogram, primarily focus on general patient conditions or a single diagnostic modality, they do not fully integrate the potential predictive value of multimodal data. This study aims to develop and validate an Imaging Score, integrating clinical and imaging features derived from whole-body ^18^F-fluorodeoxyglucose (^18^F-FDG) positron emission tomography-computed tomography (PET-CT), predicting death probability within 12 weeks from treatment initiation for refractory disease.

**MATERIALS AND METHODS:**

The development cohort comprises 254 patients from three clinical trials. Nine clinical variables and six imaging variables were assessed. After optimal subset selection through recursive Feature Elimination with cross-validation, a support vector classifier-trained machine learning model generated the Imaging Score. Validation was performed on a real-life patient cohort (n = 74). Model performance was assessed on discrimination (Harrell C-index) and calibration.

**RESULTS:**

Final prognostic features included whole-body metabolically active tumor volume, Eastern Cooperative Oncology Group performance status, visceral fat density, number of metastatic sites, body mass index, maximum standardized distance, and months since diagnosis. The Imaging Score demonstrated robust discriminative ability in both the development (C-index, 0.797) and validation (C-index, 0.714) sets, outperforming the Colon Life nomogram that tended to overestimate 12-week mortality.

**CONCLUSION:**

The Imaging Score, integrating ^18^F-FDG PET-CT imaging with clinical parameters, is an effective prognostic tool for patients with chemorefractory mCRC. This combination of imaging biomarkers with clinical factors improves discrimination, enhancing its potential for clinical decision making, patient stratification for chemorefractory treatments, and trial eligibility.

## INTRODUCTION

Colorectal cancer (CRC) is currently the third most prevalent cancer in the United States.^1^ Around 20% of patients have metastatic disease from the outset, and approximately 20%-50% of patients who present with limited-stage disease will eventually relapse.^2^ Although CRC incidence has steadily risen in developed countries, clinical outcomes have improved substantially because of early detection efforts, more aggressive surgical approaches, and new targeted therapies.^3-5^

**CONTEXT**

**Key Objective**
Can integrating clinical data with whole-body ^18^F-fluorodeoxyglucose positron emission tomography-computed tomography imaging features enhance prognostic accuracy in chemorefractory metastatic colorectal cancer (mCRC), aiding clinicians in balancing treatment efficacy and toxicity?
**Knowledge Generated**
This study introduces an innovative Imaging Score that predicts 12-week mortality by combining imaging and clinical metrics. This approach aims to provide a more holistic and accurate assessment than current prognostic tools.
**Relevance *(J.L. Warner)***
This study shows that a score taking into account routinely collected clinical and imaging variables outperforms standard tools in predicting 12-week mortality for chemorefractory mCRC, with good performance. Use of such a score may improve clinical decision making for this patient population.*
*Relevance section written by *JCO Clinical Cancer Informatics* Editor-in-Chief Jeremy L. Warner, MD, MS, FAMIA, FASCO.

However, despite these improvements, managing chemorefractory metastatic CRC (mCRC) requires a delicate balance between effectiveness and toxicity. This challenge is enhanced by the frequent deterioration of patients, which increases the probability of treatment-induced toxicities and compromises overall treatment efficacy. Considerable heterogeneity is observed within the chemorefractory mCRC patient population, and not all factors contributing to this diversity have been fully identified.^6^

Over the past few years, several new therapeutic agents have been introduced in the refractory setting, frequently offering only marginal benefit at the cost of considerable clinical and financial toxicity. Moreover, clear predictors of treatment response remain elusive.^7-9^ In this highly complex clinical setting, individual patient prognosis tools based on objective biomarkers could help clinical decision making by informing physicians about the patient's life expectancy.

In the clinical trial context, a tool for individual patient prognosis could assist in assessing the life expectancy eligibility criteria, leading to better patient selection and allowing a better interpretation of trial results. For this reason, Pietrantonio et al^10^ developed and validated the Colon Life nomogram, which aims to estimate the 12-week death probability in patients with refractory mCRC. Although performing relatively well in the development and validation cohorts, mixed results were obtained in subsequent external validations.^11-14^ Of note, the prognostic variables retained to build the nomogram (performance status, primary tumor resection, lactate dehydrogenase [LDH] value, and peritoneal involvement) mainly relate to the patient's general condition and the metastatic sites rather than the disease biology.^15-17^

^18^F-fluorodeoxyglucose positron emission tomography-computed tomography (^18^F-FDG PET-CT)–based metabolic imaging is a proven and effective tool to evaluate and track oncologic diseases. Several tumor-specific prognostic imaging features have been identified and validated.^18-21^ In mCRC specifically, it has been shown that features such as whole-body metabolic active tumor volume (WB-MATV) derived from the PET images are more specific than other clinical factors.^21^ Moreover, the patient's visceral and subcutaneous fat densities derived from the CT images from the same ^18^F-FDG PET-CT procedure also have a strong and independent prognostic value.^22^ Given these findings, we hypothesized that integrating these imaging biomarkers, possibly combined with clinical factors, would enhance prognostic accuracy and more effectively predict median overall survival (OS) compared with existing tools such as the Colon Life nomogram, which lacks detailed imaging data.

This study aims to develop and validate a prognostic model, called Imaging Score, that includes whole-body ^18^F-FDG PET-CT–based biomarkers to ultimately aid in patient selection for clinical trials and in the everyday clinical decision making of patients with chemorefractory mCRC.

## MATERIALS AND METHODS

### Patient Population

This retrospective study, approved by the Institutional Review Board at the Institut Jules Bordet (CE3408), incorporates data from two multicentric prospective phase II nonrandomized clinical trials (SoMore,^23^ RegARd-C^24^) and one single-center, single-arm, prospective, interventional, nontherapeutic clinical trial (CORIOLAN^25^), all of which were conducted within the same Belgian hospital network between 2011 and 2018. In these trials, all patients provided written informed consent before any study procedures. The trials recruited a comparable population of patients with unresectable chemorefractory mCRC with an estimated life expectancy of ≥12 weeks and Eastern Cooperative Oncology Group performance status (ECOG PS) of ≤1. A general overview of the trials can be found in the Data Supplement (Table S1). Among clinical variables collected at the time of study inclusion, only those registered in all three trials were retained to develop the Imaging Score: patient age, sex, ECOG PS, *KRAS* mutation status, primary tumor resection, tumor sidedness, BMI, months from diagnosis to study inclusion, and LDH. Next to the nine clinical variables, six variables extracted from the metabolic imaging assessment were also available, as described in the next section.

To validate the Imaging Score, a cohort of patients with chemorefractory mCRC treated according standard practice was retrospectively collected from a single center that was part of the Belgian hospital network in which the trials were carried out. Inclusion criteria were kept as similar as possible to those of the trials, comprising patients with histologically confirmed mCRC who had already undergone at least two lines of chemotherapy or monoclonal antibody treatment. Patients accrued in any of the three trials were excluded. OS was measured in months from the day of ^18^F-FDG PET-CT acquisition at the start of chemorefractory treatment until death from any cause. Treatments after PET-CT imaging included, but were not limited to, regorafenib, Lonsurf, or participation in a phase I trial, as determined by the treating physician. For the retrospective data used in this study, formal consent was not required in accordance with institutional guidelines.

### Metabolic Imaging

In each trial, a baseline whole-body ^18^F-FDG PET-CT scan was performed in strictly identical and standardized conditions following the European Association of Nuclear Medicine procedures,^26^ explained in detail in the Data Supplement.

The MATV of each lesion was defined from the ^18^F-FDG PET-CT, and baseline WB-MATV was calculated per patient as the sum of the MATV values of all target lesions without any predefined limitation. In addition, the number of metabolically active metastatic sites (M+ sites, which excludes the primary tumor site), the presence of peritoneal lesions, liver lesions, and the distance between the center of mass of the two most distant lesions normalized by the body surface area (eg, maximum standardized distance, SD_max_) were collected. SD_max_ was extracted using LIFEx freeware (Inserm, Orsay, France).^27^ Visceral fat density (in Hounsfield units) was derived from the associated CT by delineating the visceral adipose tissue on two adjacent slices at the third lumbar vertebra level.

### Statistical Methods

All statistical analyses were implemented in Python (version 3.12.0) using the pandas, scipy, and scikit-learn libraries. A statistical test was considered statistically significant with *P* value <.05. The variable distributions between the development and validation sets were compared using the Kolmogorov-Smirnov test with continuous variables and the Fisher's exact or Fisher-Freeman-Halton test with categorical variables.

For the Imaging Score, the outcome of interest was the probability of death within 12 weeks from the date of ^18^F-FDG PET-CT acquisition to confirm refractory disease. Patients lost to follow-up were censored at the time of last contact. Variables that were not recorded for more than 20% of the patient population were discarded, as well as patients with more than two missing variables or missing outcome data. Before training the Imaging Score, each variable was standardized using a Yeo-Johnson transformation to ensure the different scales and non-normal distributions of the variables did not influence the prognostic model. For handling missing data, we used mean imputation for continuous variables and the most frequent category for categorical variables, after standardizing the data. Textual categorical predictors (sex, *KRAS* mutation status, and tumor sidedness) were encoded to binomial numerical predictors.^29^ To find the most optimal subset of prognostic variables and remove redundant ones, recursive Feature Elimination with five-fold cross validation (RFECV) was applied using a support vector regressor with linear kernel. On the basis of the available training data and the type of modeling task, a support vector classifier was used to develop the Imaging Score. Hyperparameter optimization was performed using an automated grid search method to systematically test different combinations of model settings and identify the most effective ones.

In both the development and validation cohorts, the performance of the Imaging Score was quantified using calibration, for example, calibration plots and Hosmer and Lemeshow test, and discrimination, for example, Harrell C-index with 95% bootstrap CI. To compare performance of the Imaging Score and the Colon Life nomogram in the development and validation cohorts, the 12-week death probability was also calculated according to the original publication of the nomogram, as presented in the Data Supplement. Patients with missing values for primary tumor resection, LDH, ECOG PS, or peritoneal metastasis were excluded from the Colon Life analysis.

## RESULTS

### Patient Population

Baseline patient and disease characteristics are summarized in Table 1. In total, 254 patients with mCRC were available for model development, with 245 deaths from any cause, of which 50 (19.7%) were within 12 weeks. Thirty-one patients were excluded from the analysis because of missing variables and/or outcomes. Median OS for the development cohort was 6.7 (range, 0.2-55.6) months. To evaluate the performance of the Colon Life nomogram, 126 patients could be included from the development data set.

**TABLE 1.** Baseline Patient and Disease Characteristics of the Development and Validation Cohort

Characteristic	Development Cohort (n = 254)	Validation Cohort (n = 74)	*P*
Age, years, Median (IQR)	65 (59-71)	60 (55-71)	**.005**
Sex, No. (%)			.51
Female	112 (44)	31 (42)	
Male	142 (56)	43 (58)	
ECOG PS, No. (%)			**.02**
0	125 (49)	21 (28)	
1	129 (51)	43 (58)	
2	0	9 (12)	
3	0	1 (1)	
BMI, median (IQR)	25 (23-28)	24 (21-29)	.72
Visceral fat density (HU), median (IQR)	–91 (–97 to –82)	–90 (–95 to –83)	.81
Months since diagnosis, median (IQR)	36 (23-59)	21 (15-52)	**<.001**
Primary tumor resection, No. (%)			.06
Yes	163 (64)	53 (72)	
No	34 (13)	21 (28)	
Missing	57 (23)	0	
*KRAS*, No. (%)			.27
Wild	110 (43)	24 (32)	
Mutant	142 (56)	44 (59)	
Missing	2 (1)	6 (9)	
LDH, U/L			**<.001**
Median (IQR)	422 (325-656)	393 (294-724)	
Missing	128 (50)	3 (4)	
WB-MATV, cm^3^, median (IQR)	165 (52-473)	131 (52-507)	<.59
SD_max_, cm, median (IQR)	13 (8-20)	32 (21-45)	**<.001**
Metastatic sites, No. (%)			.14
1	63 (25)	26 (35)	
2-4	176 (69)	46 (62)	
≥5	15 (6)	2 (3)	
Peritoneal metastasis, No. (%)			.09
Yes	51 (20)	8 (11)	
No	203 (80)	66 (89)	
Liver metastasis, No. (%)			1.00
Yes	194 (76)	57 (77)	
No	60 (24)	17 (23)	

NOTE. Bold values indicate significant *P* values.

Abbreviations: ECOG PS, Eastern Cooperative Oncology Group performance status; LDH, lactate dehydrogenase; SD, standard deviation; WB-MATV, whole-body metabolic active tumor volume.

A routine cohort of 74 patients with chemorefractory mCRC was collected for validation, 72 of whom died, including 14 (19.4%) within 12 weeks. Median OS for the validation cohort was 4.9 (range, 0.8-23.3) months, which was significantly lower than the development cohort (*P* = .003). In addition, compared with the development cohort, the validation cohort was younger (*P* = .005), had a higher ECOG PS (*P* = .02), had a shorter diagnosis-to-chemorefractory disease interval (*P* < .001), and had a higher tumor dissemination (SD_max_, *P* < .001), but a lower LDH (*P* < .001). In the validation cohort, 71 patients had complete data to compare the performance of the Imaging Score with the Colon Life nomogram.

### Selected Features

**FIG 1.**
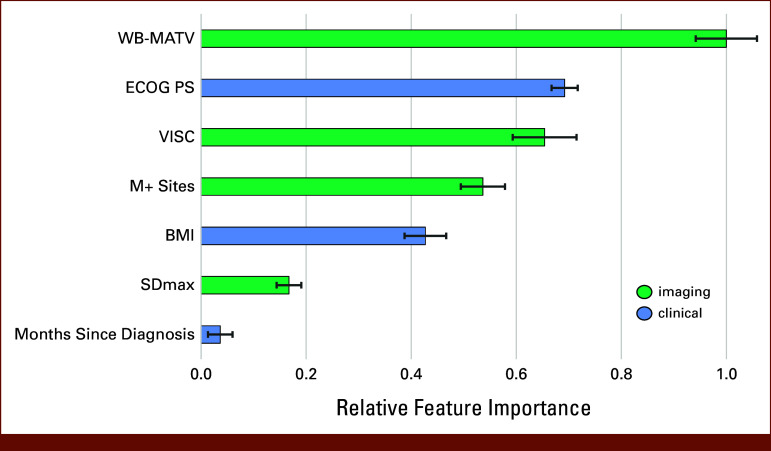
Mean relative feature importance in the final Imaging Score, with standard deviations obtained in 100 bootstraps. ECOG PS, Eastern Cooperative Oncology Group performance status; SD, standard deviation; VISC, visceral fat density; WB-MATV, whole-body metabolic active tumor volume.

Seven variables were consistently selected as the most optimal subset of prognostic variables when applying 5-fold RFECV and composed the Imaging Score: WB-MATV, ECOG PS, visceral fat density (VISC), M+ sites, BMI, SD_max_, and months since diagnosis, in order of relative importance as illustrated in Figure 1. Variables that were not selected were age, sex, *KRAS* mutation status, primary tumor resection, and presence of peritoneal or liver lesions. Tumor sidedness and LDH had to be discarded from model development because of more than 20% missing values across the cohort, despite the latter showing a strong correlation with OS.

### Imaging Score Performance and Validation

**FIG 2.**
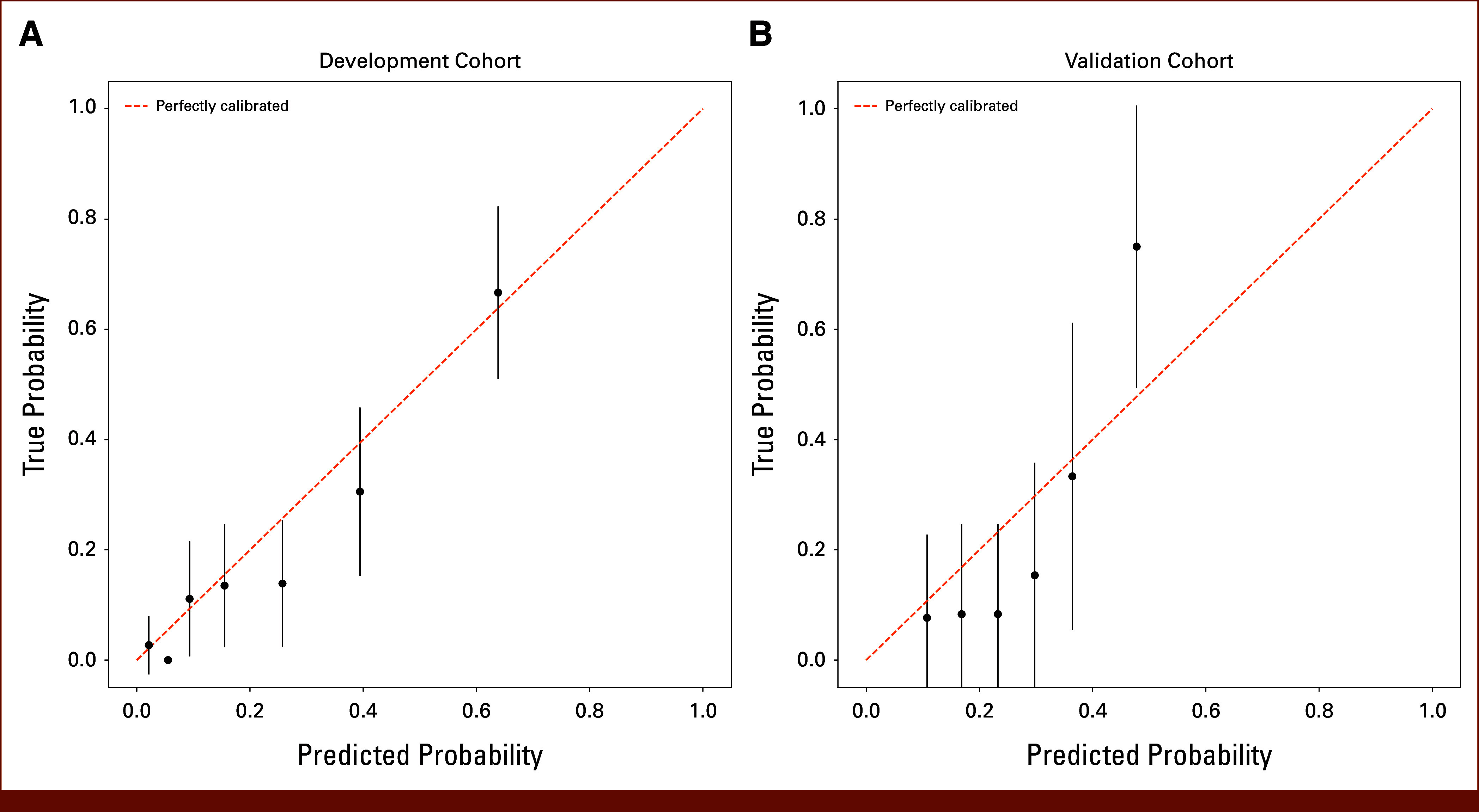
Calibration curves for the Imaging Score are presented for both the (A) development and (B) validation cohorts. Predicted probabilities were stratified into equally sized subgroups. Each subgroup's average 12-week predicted probability of death (*x*-axis) is plotted against the observed proportion of 12-week deaths (*y*-axis). Vertical lines indicate 95% CIs of the estimates.

The Imaging Score demonstrated a good discriminative ability, evidenced by a Harrell C-index of 0.797 (95% CI, 0.733 to 0.860) for the development set and 0.714 (95% CI, 0.713 to 0.714) for the validation set. In Figure 2, the calibration curves for both the development set and the validation set reveal predictions aligning with observations, as indicated by the nonsignificant Hosmer-Lemeshow statistics in both sets.

The trained Imaging Score and a data preprocessing pipeline have been made publicly available on GitHub. The repository can be accessed online on GitHub.^30^

### Colon Life Nomogram

**FIG 3.**
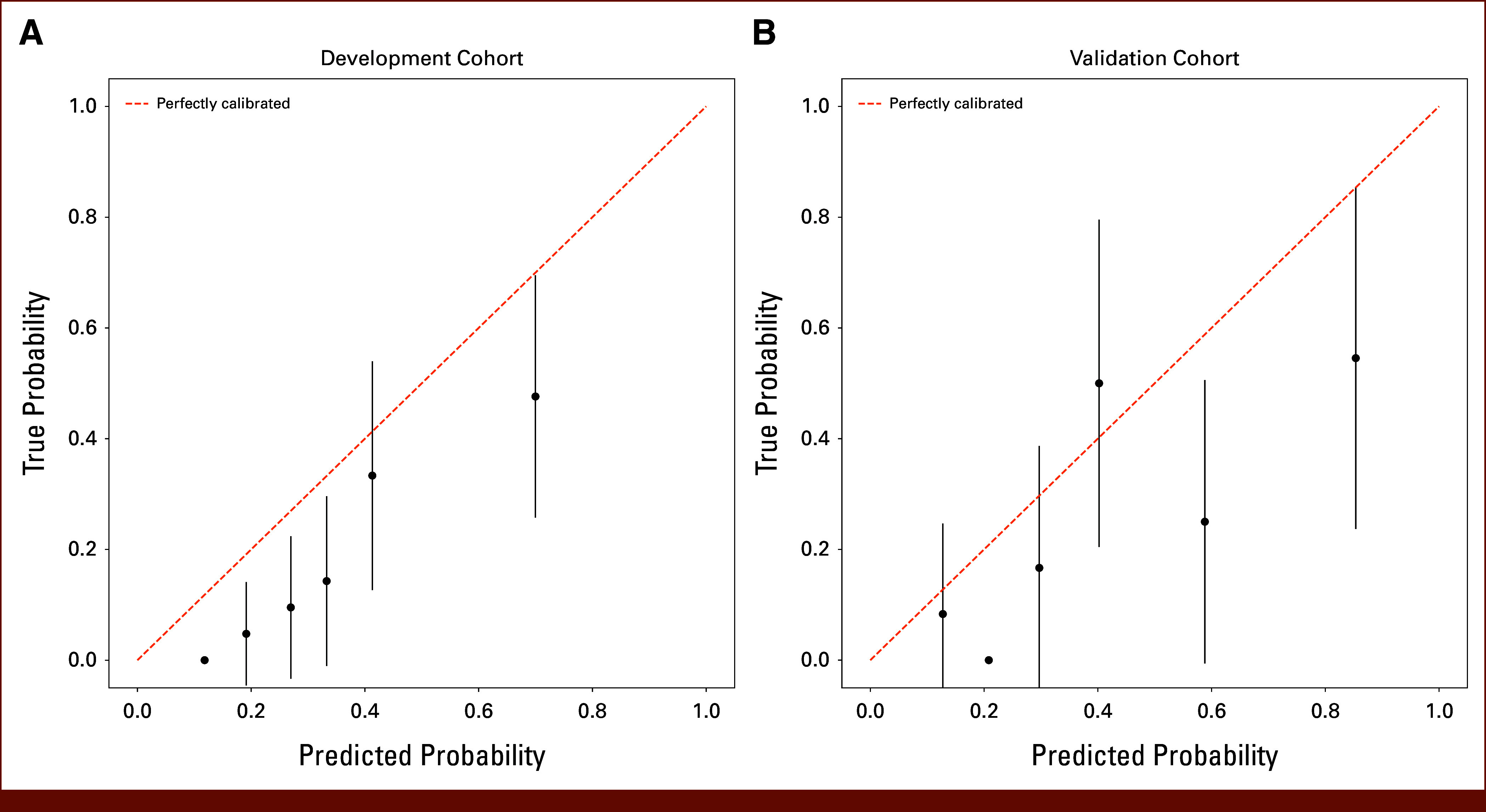
Calibration curves for the Colon Life nomogram are presented for both the (A) development and (B) validation cohorts. Predicted probabilities were stratified into equally sized subgroups. Each subgroup's average 12-week predicted probability of death (*x*-axis) is plotted against the observed proportion of 12-week deaths (*y*-axis). Vertical lines indicate 95% CIs of the estimates.

The Colon Life Score displayed lower Harrell C-index values than the Imaging Score, with a C-index of 0.653 (95% CI, 0.651 to 0.654) in the development set and 0.646 (95% CI, 0.642 to 0.650) in the validation set. The calibration assessment, illustrated in Figure 3, highlighted a systematic deviation from perfect calibration, indicating a consistent overestimation of observed probabilities, which was not observed in the Imaging Score. Both the development and validation sets yielded significant Hosmer-Lemeshow test results (*P* value = .0013 and .0008, respectively).

## DISCUSSION

Selecting patients with chemorefractory mCRC for further treatments or clinical trial inclusion is challenging for physicians, as they must balance toxicity and efficacy of treatment on the basis of a growing availability of data and potential biomarkers.^31^ The use of objective tools, such as the Colon Life nomogram by Pietrantonio et al,^10^ can help clinicians make more informed decisions to avoid unnecessary treatments, reducing the risk of futile treatments where patients may be subjected to interventions that offer no real benefit, and by providing a standardized approach to determining the 12-week survival landmark commonly used in oncology trials. However, our findings suggest that the nomogram may overestimate death probability, consistent with other external validations.^11-13^

In our study, we have explored the potential benefits of incorporating parameters from both ^18^F-FDG PET-CT imaging and clinical evaluation into a machine learning model to predict the probability of death within 12 weeks from the start of treatment in a population of patients with advanced chemorefractory CRC. The discriminative ability of the subsequent Imaging Score, in which both clinical and imaging parameters were retained, proved effective in both the development and validation sets (Fig 2). The composition of the development set, comprising patients with diverse treatments from two multicenter trials and one single-center trial, likely contributed to the establishment of a robust model. Despite statistically significant differences in several features and OS between the development and validation sets (Table 1), the performance of the Imaging Score generalized relatively well.^32,33^

The model's principal driver was WB-MATV, a robust ^18^F-FDG PET-CT–based biomarker that has already undergone prospective validation in patients with chemorefractory mCRC.^34^ Additionally, VISC^22^ and M+ sites^35^ in patients with mCRC, along with SDmax in lymphoma^36^ and esophagal squamous cell carcinoma,^37^ have individually demonstrated prognostic value. Clinical features retained in the Imaging Score were ECOG PS, already present in the Colon Life Score, months since diagnosis, and BMI. The latter was not considered in the Colon Life Score because of data collection limitations.^10^ This addition is supported by the recognized prognostic relevance of BMI, as highlighted in a pooled analysis by Renfro et al.^17^

Interestingly, the Imaging Score did not retain the *KRAS* mutation status (which is an established prognostic and predictive parameter in mCRC), suggesting that, especially in the refractory setting, a combination of parameters objectively quantifying the general patient condition (VISC, BMI, and ECOG PS) and the disease burden (WB-MATV, M+ sites, and SD_max_) might have more prognostic power than certain specific molecular biomarkers.

In comparison with studies that focus on a single type of feature (such as general patient condition^10^ or using a single assessment modality, such as radiomics studies^38^), the Imaging Score integrated multiple features from different modalities (ie, clinical evaluation, and molecular and anatomic imaging) with minimal added complexity, as it relies on a single additional examination that provides valuable information on both the patient's nutritional status and the tumor's aggressiveness. Additional improvements could be made by adding parameters from the Colon Life Score (LDH could not be incorporated because of too many missing values) and other promising biomarkers from sources beyond imaging that were not included in this study because of data limitations. For example, the integration of quality of life used by Hamers et al^12^ to enhance the Colon Life Score and the inclusion of cfDNA^39^ could contribute to a comprehensive and improved prognostic model. Neutrophil-to-lymphocyte ratio has also been shown to be a potential predictive biomarker for survival in patients with mCRC.^40^

Our study has some limitations. The manual segmentation of the WB-MATV is a time-consuming process, and future studies could benefit from implementing deep learning algorithms to streamline this task. Using the PERCIST threshold for segmentation, while conventional for target lesion definition rather than MATV, introduces the potential to overlook lesions with low uptake, leading to the exclusion of some patients from our data set.^41^ Furthermore, ensuring consistent and reliable results of our Imaging Score in other centers would require the standardization or harmonization of PET image acquisition.^42,43^ Regarding the validation cohort, while it was representative of real-life clinical practice and differed from the development cohort, it was relatively limited in size. Additionally, since the validation cohort stemmed from a hospital that participated in the clinical trials from which the development cohort was derived, there is a potential overlap in clinical practices and patient management strategies, which may reduce the generalizability of the findings. Therefore, further external validation in independent hospital networks is crucial to assess the robustness and applicability of the Imaging Score across diverse clinical settings, which can be facilitated through its availability on GitHub, allowing for further testing. Larger, multicenter studies will be necessary to confirm the model's utility and to ensure that it can be effectively implemented in a wider range of health care environments.^44^

In conclusion, our approach, prioritizing imaging biomarkers in combination with general clinical parameters, proves effective in the development of a well-performing prognostic tool for patients with chemorefractory mCRC. This study highlights the importance of a balanced and comprehensive model, integrating both patient-specific and disease-related factors, mainly derived from ^18^F-FDG-PET-CT, a single diagnostic procedure. By improving prognostic accuracy, this model has the potential to impact clinical decision making, enabling better patient stratification and refining trial eligibility for patients with chemorefractory mCRC.

## Data Availability

A data sharing statement provided by the authors is available with this article at DOI https://doi.org/10.1200/CCI-24-00207.
